# Supplementary material for: PolySUMOylation of PCNA and Rad52 restricts centromeric recombination in fission yeast
Source: Nat Commun. 2025 Dec 2;16:10837. doi: 10.1038/s41467-025-65862-1 (PMC12672585; doi:10.1038/s41467-025-65862-1)
Supplement: Supplementary file 1 — Supplementary Information [file 41467_2025_65862_MOESM1_ESM.pdf]

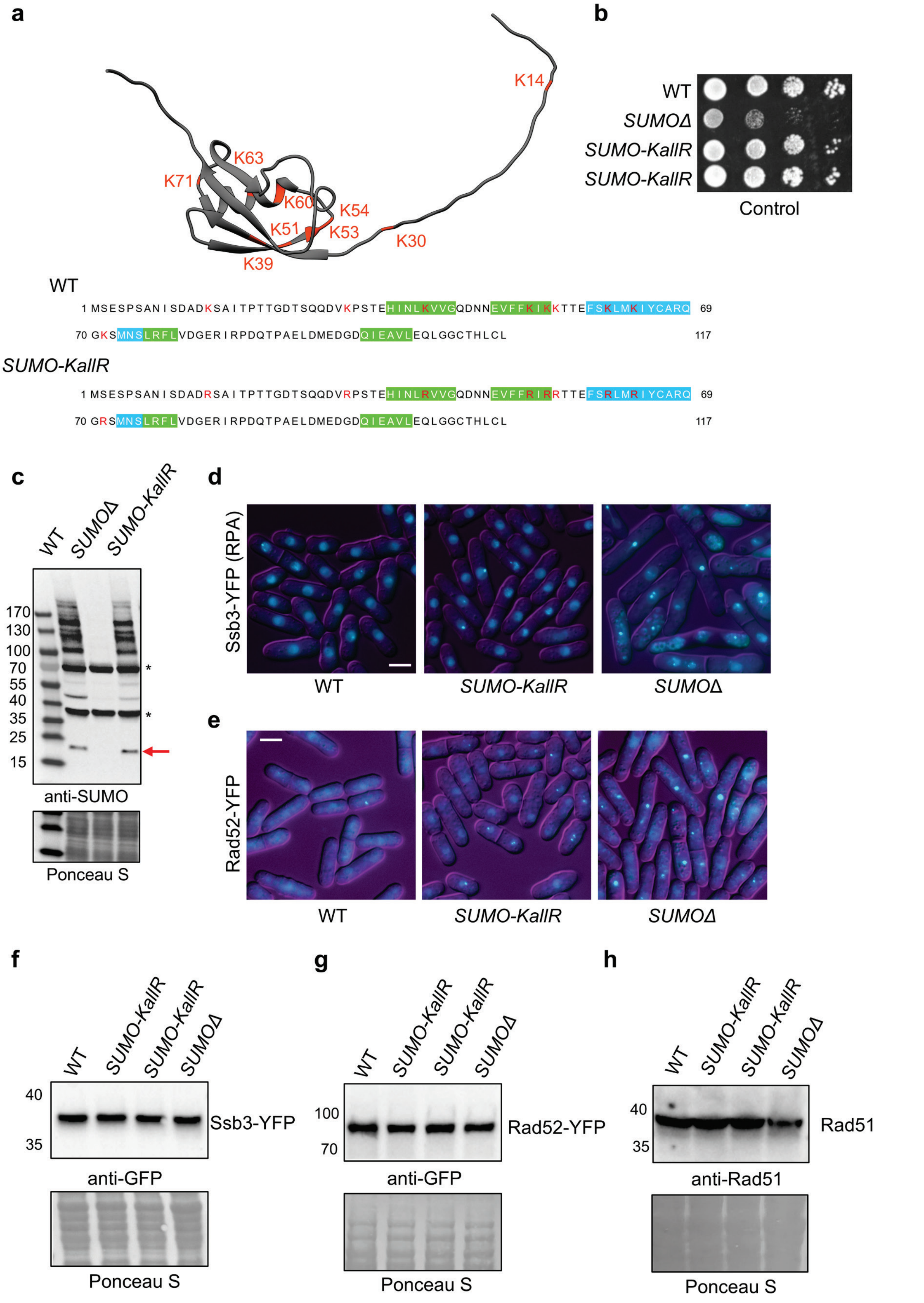

Supplementary Figure 1: Loss of SUMO chains leads to elevated replication stress and spontaneous DNA damage (related to Fig. 1). a A schematic model depicting the SUMO-KallR protein molecule. Nine lysine

residue positions, including key K_{14} and K_{30} necessary for chain formation, mutated to arginines (R) in the *SUMO-KallR* allele, are marked red, while β-strands and α-helices are highlighted in green and blue, respectively, in amino acid sequences. The model was made using AlphaFold Server. **b** Cell growth assay of indicated strains. Ten-fold serial dilution of exponential cultures were dropped on YES agar plates **c** Expression of SUMO conjugates in indicated strains on anti-Pmt3 Western blot. mono-SUMO moieties are indicated with a red arrow; * indicates unspecific signal. Ponceau S stained blot mark protein loading onto gel. Molecular weight marked alongside marker in kDa. **d** Example of fluorescence images of logarithmically growing cells expressing RPA containing the $Ssb3^{RPA3}$ subunit endogenously tagged with YFP in indicated strains. Scale bar = 5 µm. **e** Example of fluorescence images of logarithmically growing cells expressing endogenous Rad52-YFP in indicated strains. Scale bar = 5 µm. **f-h** Expression of Ssb3-YFP, Rad52-YFP and native Rad51, respectively, analysed by Western blot with anti-GFP or anti-Rad51 antibody of total protein extracts isolated from exponentially growing cultures of indicated strains. Ponceau S stained blots were added to show equal amounts of total proteins loaded onto the gel after isolation from individual strains.

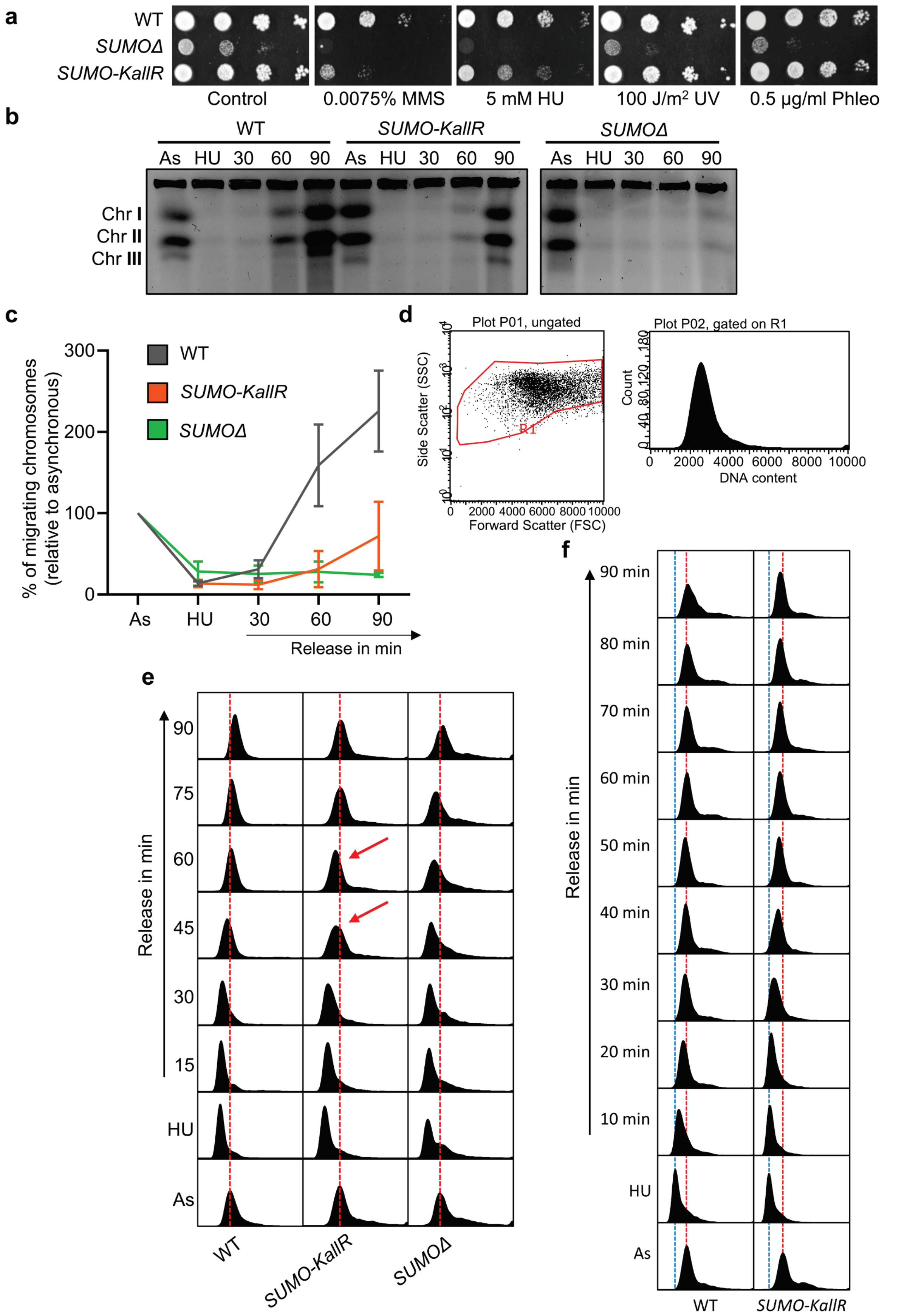

Supplementary Figure 2: SUMO chains enable completion of disturbed replication and control the timing of DNA repair. a Cell growth assay of indicated strains. Ten-fold serial dilution of exponential cultures

were dropped on YES agar plates containing appropriate genotoxic drugs in specified concentration: MMS, methyl methanesulfonate; HU, hydroxyurea; Phleo, phleomycin or exposed to UV-C light. **b** Representative images of intact chromosome migration during pulsed field gel electrophoresis (PFGE). Logarithmically growing cells (As, asynchronous cells) of indicated strains were exposed to 20 mM HU for 4 h (HU time point) and then released into fresh, HU-free, rich YES medium at 30°C to monitor recovery of chromosomes 30, 60 and 90 min after drug removal. **c** % quantification of chromosomes migrating into the gel after release from HU block relative to their asynchronous profile. Values are means of 3 independent biological replicates ± SD. **d** Gating strategy for conducted flow cytometry analysis. Left panel: ungated SSC-FSC plot; right panel: gated histogram based on SSC-FSC plot. **e** Flow cytometry analysis of DNA content in indicated strains. Logarithmically growing cells (As, asynchronous cells) were exposed to 20 mM HU for 4 h (HU time point) and then released into fresh, HU-free, rich YES medium at 30°C to monitor S-phase progression at the indicated time (min) after release. Red arrows indicate the delay in S phase progression of *SUMO-KallR* mutant compared to WT strain. Red dashed line marks G2 phase in asynchronous cells. **f** Flow cytometry analysis of DNA content in indicated strains. Logarithmically growing cells of WT and *SUMO-KallR* (As, asynchronous cells) were exposed to 20 mM HU for 4h (HU time point) and then released into fresh, HU-free, rich YES medium at 30°C to monitor S-phase progression in the 10 min intervals. Blue dashed line marks S-phase synchronisation, red dashed line G2 content.

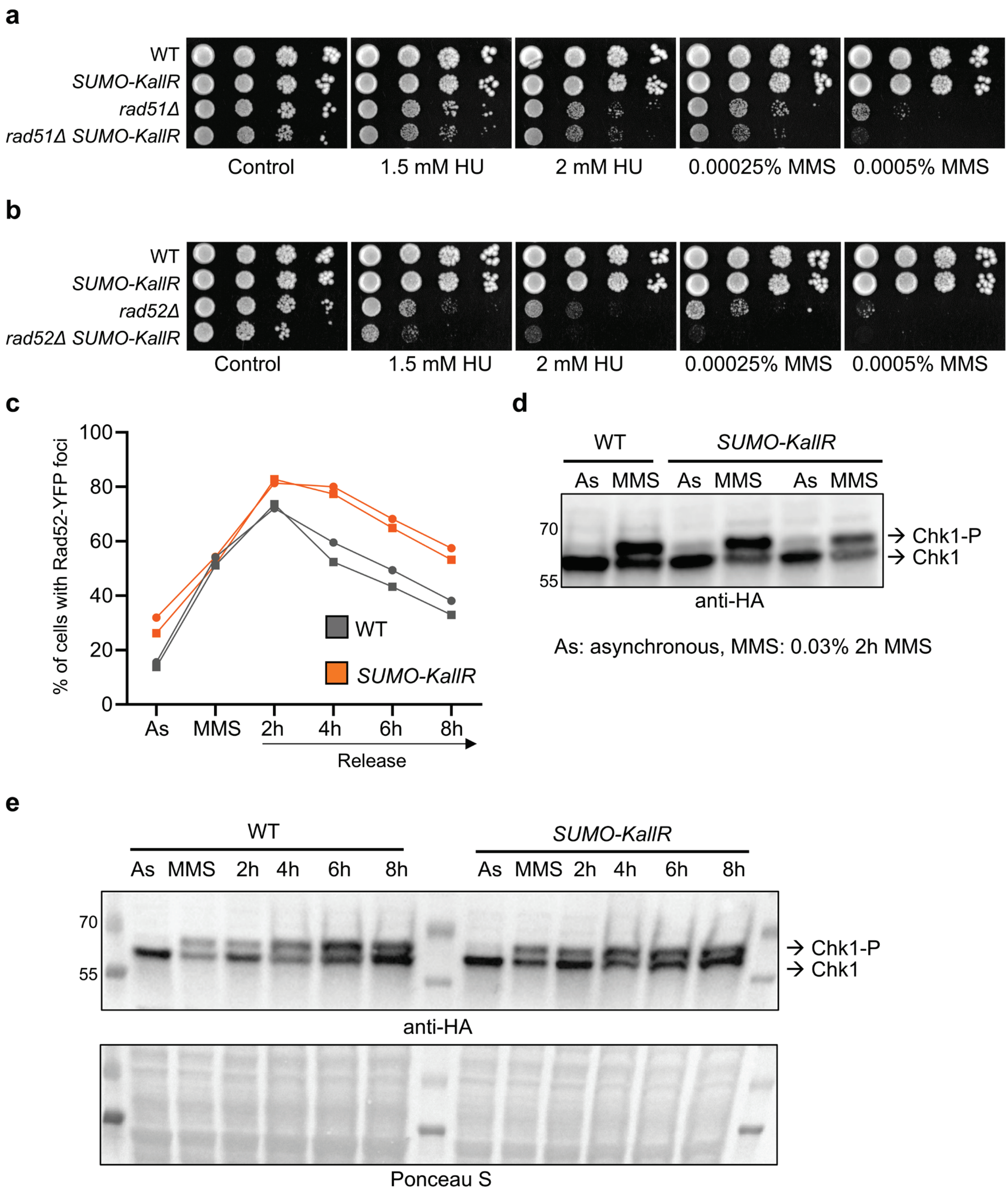

Supplementary Figure 3: SUMO chains control the timing of DNA repair. a-b Drop dilution assay of indicated strains (performed as in **Fig. S2a**) to indicated drugs. **c** The dynamics of formation and resolution of recombination repair centers in response to MMS-induced DNA damage in indicated strains. Logarithmic cells expressing the endogenous Rad52-YFP fusion protein (As, asynchronous cells) were exposed to 0.03% MMS for 2 h (MMS time point) and then released into fresh medium. Microscopy of Rad52-YFP was conducted in two-hour intervals up to 8 hours after drug removal in two biological replicates for each strain. The percentage of cells forming Rad52-YFP foci at all time-points examined was calculated. **d** The cell cycle DNA damage checkpoint activation in response to MMS in indicated strains. Phosphorylation of Chk1 kinase (Chk1-P) in response to 0.03% MMS for 2 h was confirmed by Western blot with anti-HA antibody of total protein extracts isolated from exponentially growing and

MMS-exposed cultures. The experiment was repeated twice. Molecular weight marked alongside the marker in kDa. **e** The dynamics of the cell cycle DNA damage checkpoint inactivation after MMS treatment in indicated strains. Logarithmic cells expressing the endogenous Rad52-YFP fusion protein (As, asynchronous cells) were exposed to 0.03% MMS for 2 h (MMS time point) and then released into fresh medium as in **c** Samples were collected in two-hour intervals up to 8 hours after drug removal and phosphorylation of Chk1 kinase (Chk1-P) was shown by Western blot with anti-HA. Molecular weight marked alongside the marker in kDa.

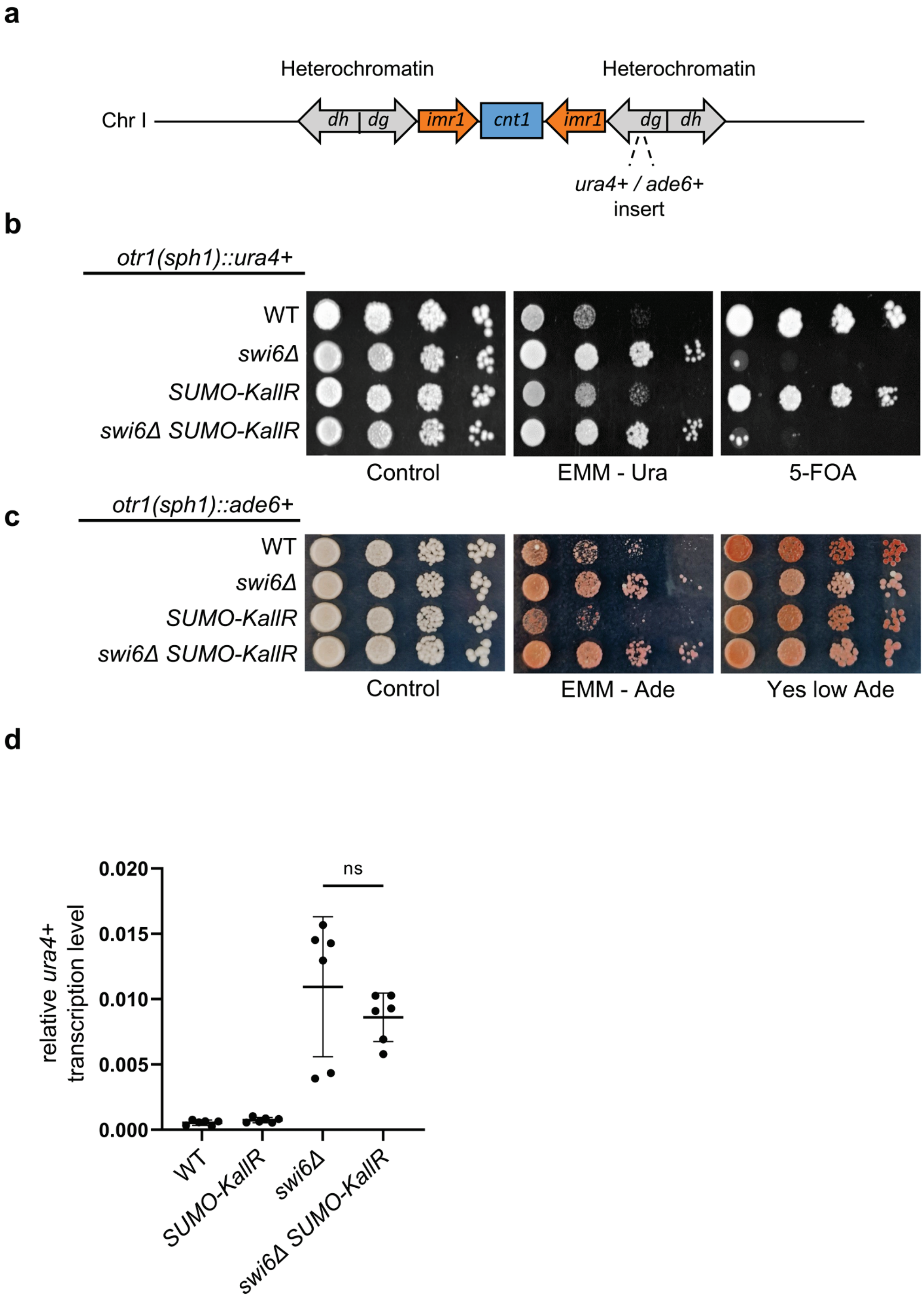

Supplementary Figure 4: SUMO chains do not impact silencing in the centromeric locus. a A schematic diagram depicting insertion of *ura4+* or *ade6+* reporter gene between *otr1* inverted repeats located in

the centromere *cen1* of the fission yeast chromosome I. Expression of functional products of these genes from this locus is suppressed in normal physiological conditions due to the heterochromatin silencing. **b** Silencing assay of indicated strains. Ten-fold serial dilution of exponential cultures were dropped on EMMg agar plates lacking uracil or containing 5-FOA in specific concentration. **c** Silencing assay of indicated strains. Ten-fold serial dilution of exponential cultures were dropped on EMMg agar plate lacking adenine and YES agar plate containing adenine in lower concentration. **d** Relative *ura4+* transcription level in indicated strains analysed by qPCR using relevant primers (listed in Table S2). *ura4+* enrichment was calculated over actin values. Dots represent values obtained from 3 independent biological experiments with 2 technical repeats. Error bars show the SD about the mean values. Two-sided Student's *t*-test was used to calculate the *p* values (not significantly different).

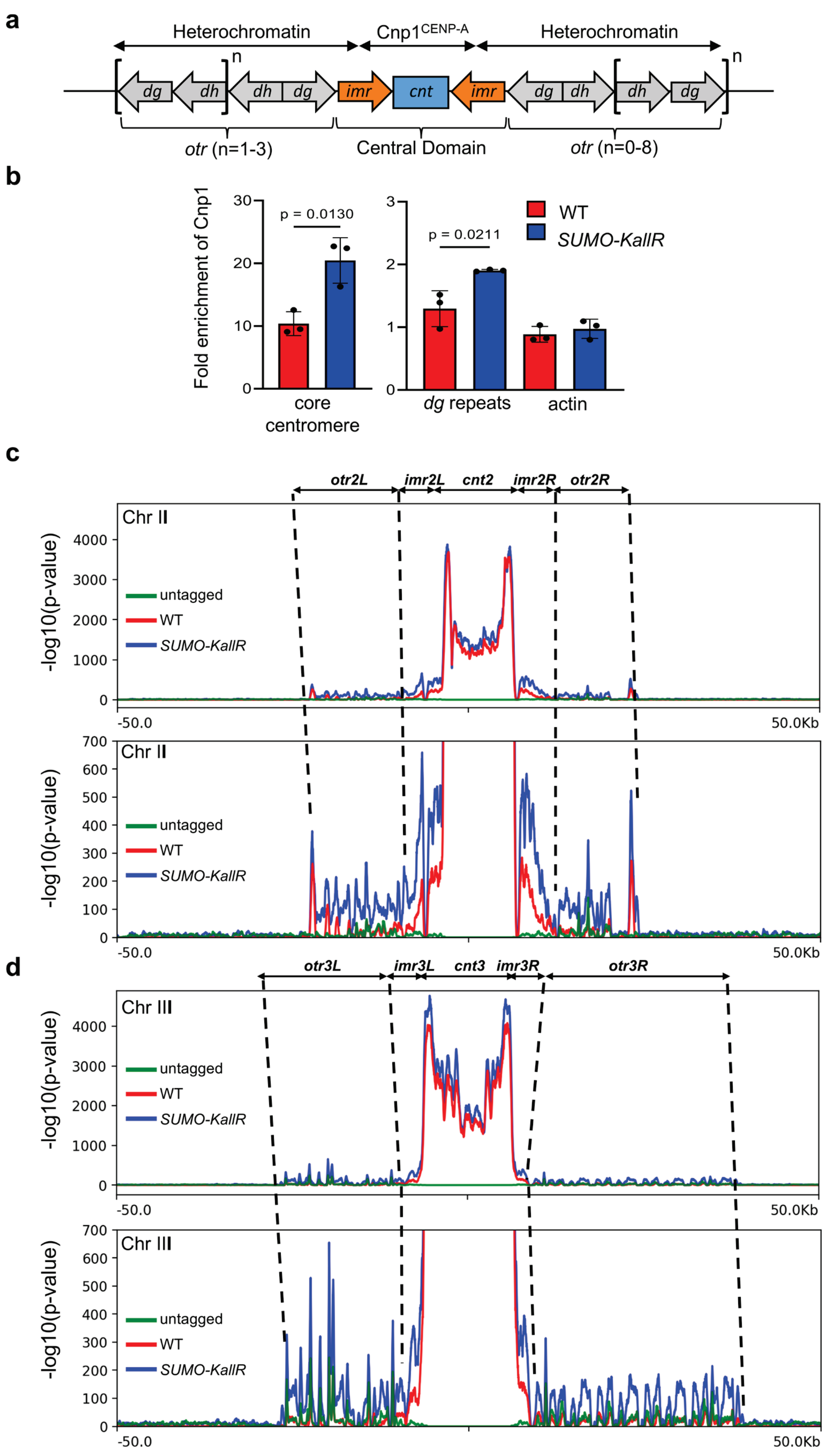

Supplementary Figure 5: Deficiency of SUMO chains leads to centromere disruption (related to Fig. 2).
a A schematic diagram depicting DNA structure of fission yeast centromeres. All three centromeres

possess the same overall structure where two outer repeats region (*otr*, gray arrow boxes) surround a central domain: core (*cnt*, blue rectangle box) flanked by two innermost repeat regions (*imr*, orange arrow boxes). The *otr* contain identical elements between the three chromosomes, although the number of repetitions (*dh/dg*) varies. The *otr* repeats together with part of the *imr* are covered with heterochromatin. The central domain (*cnt* region and part of the *imr*) is characterized by the presence of the centromeric histone variant Cnp1$^{CENP\text{-}A}$ and corresponds to the region where kinetochore is assembled. **b** Binding of CFP-Cnp1 to centromere locus in indicated strains analysed by ChIP-qPCR using anti-GFP antibody and relevant primers (listed in Table S2). Enrichment for actin locus was presented as unrelated control. Dots represent values obtained from 3 independent biological repeats. Error bars show the SD about the mean values. Two-sided Student's *t*-test was used to calculate the *p* values. **c-d** Calibrated ChIP-seq profiles of chromosome II (**c**) and III (**d**) CFP-Cnp1 binding in indicated strains. Logarithmic cultures of indicated strains were mixed with budding yeast calibrator aliquot (exponential culture of Scc1-Pk) and extracted DNA was used to construct DNA libraries and for NGS analysis. The range of central domain and *otr* region are designated with black dashed lines. The central part of *cnt* was set as 0. For each chromosome the upper panel presents locus around the centromere with values on Y-axis, that present -log10(p-value) set up to 4000 to cover the signal from the core of centromere, whereas bottom panel scale on Y-axis was set up to 700.

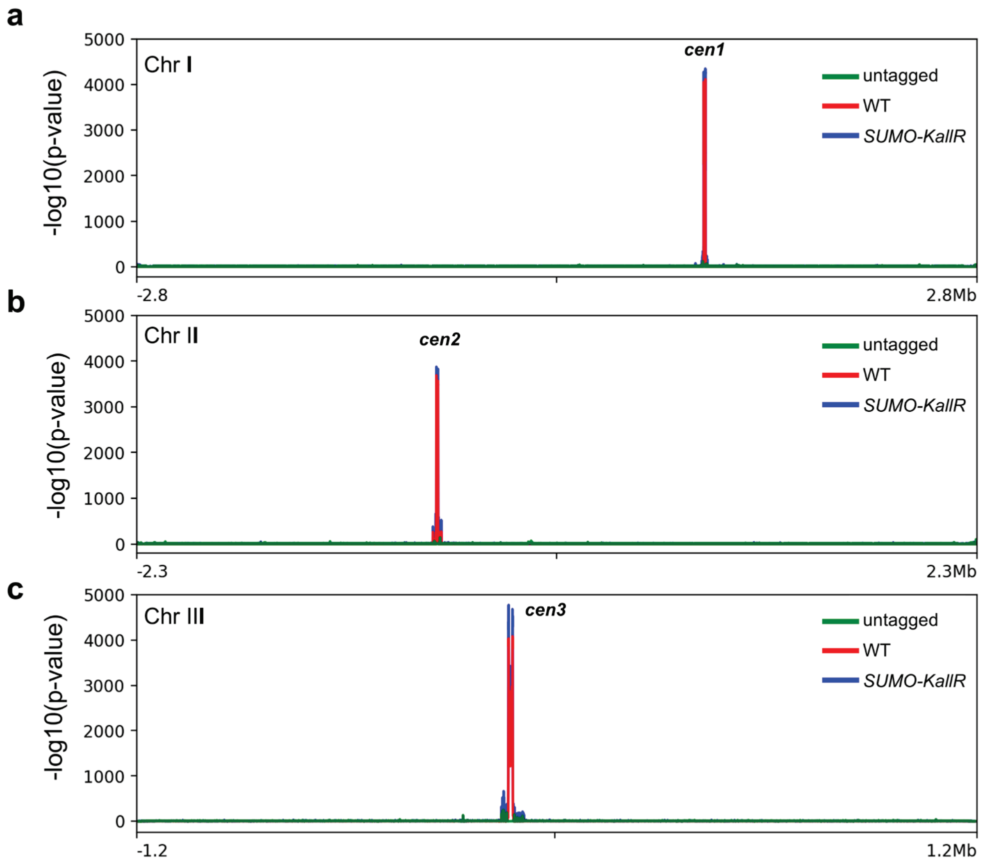

Supplementary Figure 6: Deficiency of SUMO chains leads to centromere disruption (related to Fig. 2). **a-c** Calibrated ChIP-seq profiles of whole chromosome I (**a**), II (**b**) and III (**c**) CFP-Cnp1 binding in indicated strains. Logarithmic cultures of indicated strains were mixed with budding yeast calibrator aliquot (exponential culture of Scc1-Pk) and extracted DNA was used to construct DNA libraries and NGS

analysis. The range of central domain and otr region are designated with black dashed lines. The central part of *cnt* was set as 0. For each chromosome the panel presents locus around the centromere with values on Y-axis, that present -log10(p-value) set up to 5000 to cover the signal from the core of centromere.

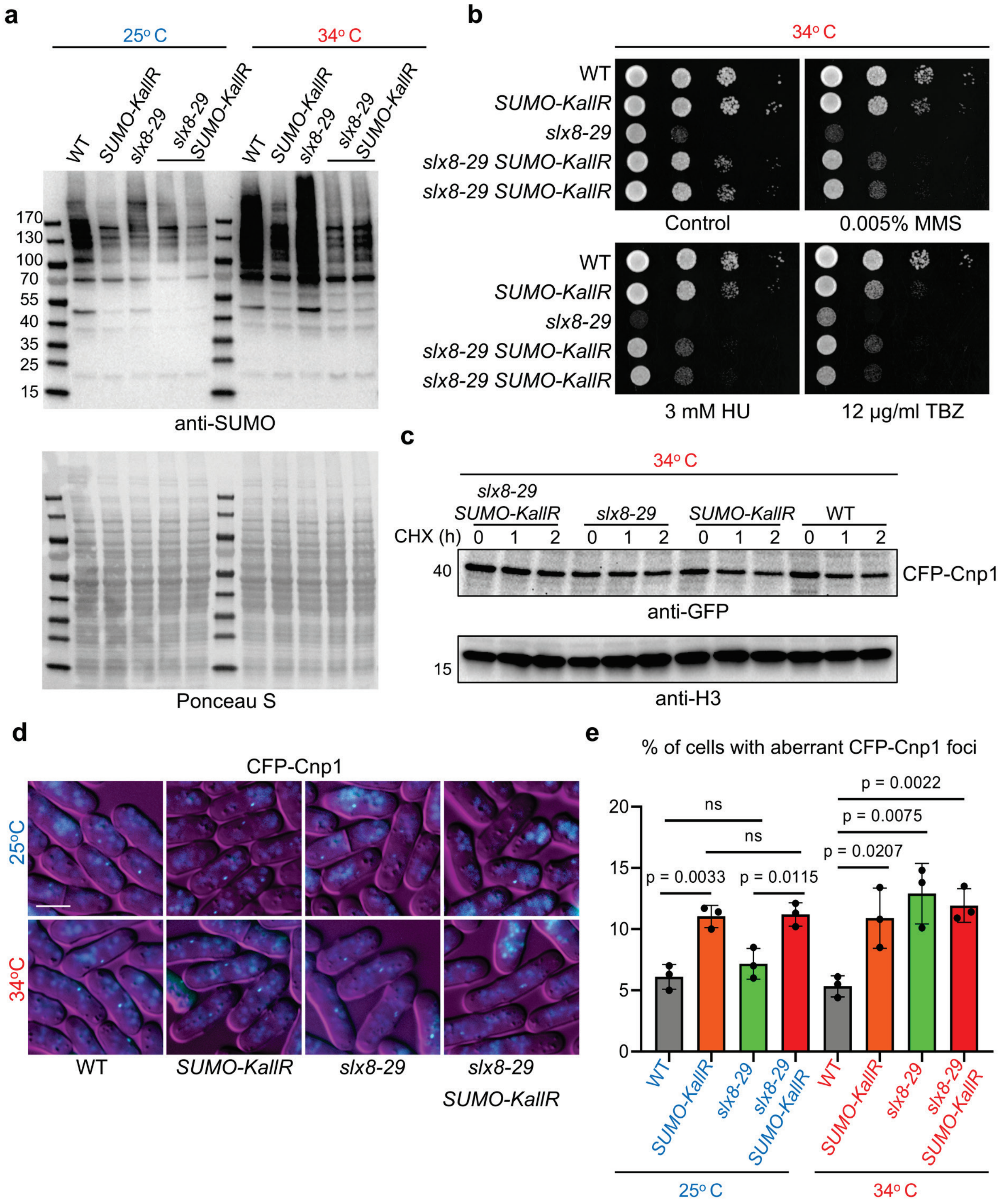

Supplementary Figure 7: Slx8 STUbL does not play a major role in Cnp1^{CENP-A} regulation in *S. pombe*. a Expression of SUMO conjugates in indicated strains and temperatures (blue – permissive, 25°C, red – restrictive 34°C). Total protein extracts isolated from exponentially growing cultures were analysed by Western blot with anti-Pmt3 antibody. Ponceau S stained blots were added to show equal amounts of

total proteins loaded onto the gel. Molecular weight marked alongside the marker in kDa. **b** Drop dilution assay of indicated strains (performed as in **Fig. S2a**) to indicated drugs. **c** Stability of CFP-Cnp1 in indicated strains. Exponentially growing cells of the WT strain and selected mutants were treated for 1 or 2 h or not (timepoint 0) with cycloheximide (CHX) to a final concentration of 250 µg/ml at 34°C (a cycloheximide chase experiment). At selected time points, samples were collected, and total protein extracts were prepared using the standard TCA method. Samples were then subjected to Western blotting using anti-GFP for CFP-Cnp1 detection and anti-H3 antibodies to assess loading. Molecular weight marked alongside the marker in kDa. **d** Examples of live cell imaging of logarithmically growing cells expressing endogenous CFP-Cnp1 fusion protein in indicated strains. Scale bar = 5 µm. **e** % quantification of cells forming aberrant Cnp1 foci in indicated strains. Dots represent values obtained from 3 independent biological experiments. In all, at least 500 cells were analysed for each strain. Error bars show the SD about the mean values. Two-sided Student's *t*-test was used to calculate the *p* values.

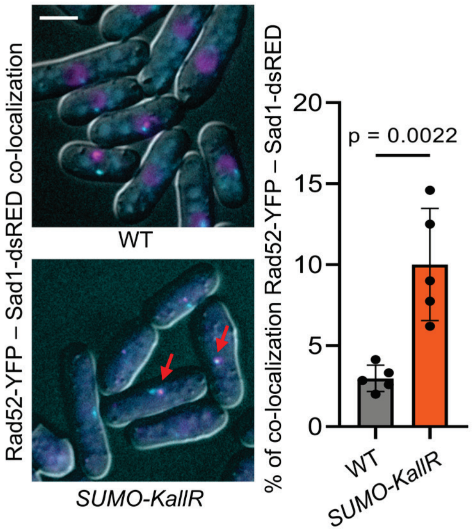

Supplementary Figure 8: Increased Rad52 co-localization with spindle pole body Sad1 (related to Fig. 3). **a** Example of DIC and merged fluorescence images of logarithmic *SUMO-KallR* cells expressing endogenous Rad52-YFP and Sad1-dsRed. Red arrows indicate co-localization between two proteins. Scale bar = 5 µm. **b** % quantification of co-localization between Rad52-YFP and Sad1-dsRed in indicated strains. Dots represent values obtained from 5 independent biological experiments. In every experiment 200-300 cells were analysed for each strain. Error bars: SD about the mean values. Two-sided Student's *t*-test was used to calculate the *p* values.

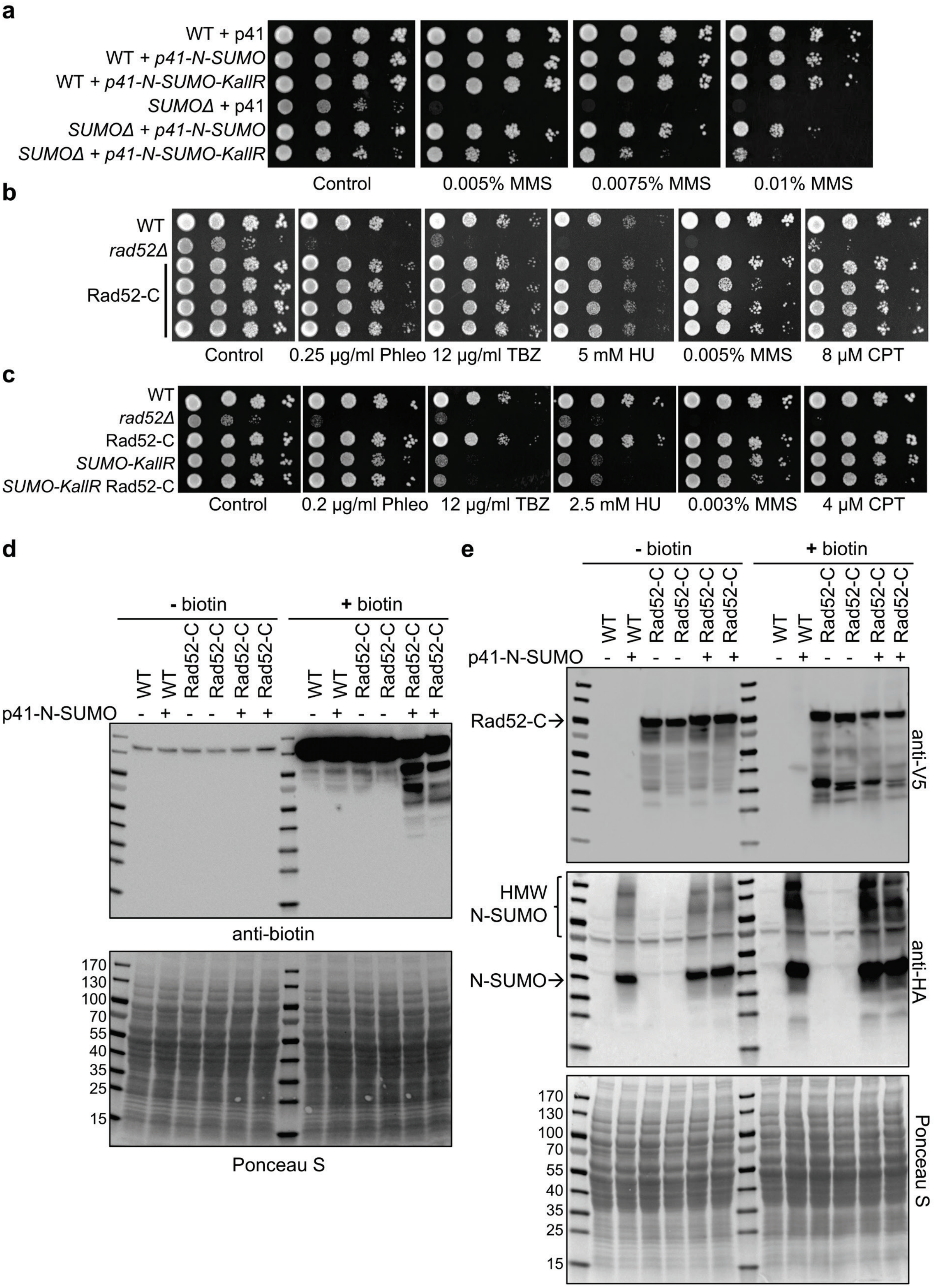

Supplementary Figure 9: Analysis of Rad52- and SUMO-dependent interactome (related to Fig. 4). a Complementation assay of p41-NTurbo-SUMO and p41-NTurbo-SUMO-KallR functionality in indicated transformants. Empty vector pREP41 was designated as p41. Ten-fold serial dilution of exponential cultures were dropped on YES agar plates containing specific MMS concentration. **b-c** Analysis of

functionality of Rad52-Split-TurboID by drug sensitivity assay compared indicated strains. Ten-fold serial dilution of exponential cultures were dropped on YES agar plates containing Phleo, TBZ, HU, MMS or CPT in specific concentration. **d** Proximal biotinylation assay of proteins by Split-TurboID in indicated transformants and conditions. Western blot analysis of total cell extracts prepared from a control untagged (WT) and the Rad52-12Pk-CTurbo (Rad52-C) strains transformed with an empty vector or 6HA-Nturbo-SUMO (N-SUMO) bearing plasmid was performed using anti-biotin antibody (upper panel). A Ponceau S stained blot (lower panel) was added to show equal amounts of total proteins loaded onto the gel after isolation from an individual transformant. Molecular weight marked alongside the marker in kDa. **e** Expression of Rad52-12Pk-CTurbo (Rad52-C) and 6HA-NTurbo-SUMO (N-SUMO) in indicated conditions. Western blot analysis with anti-V5 (upper panel), and anti-HA (middle panel) antibody, respectively, was performed for total protein extracts isolated from exponentially growing cultures of indicated transformants. High molecular weight (HMW) SUMO-conjugates were put in bracket. The blot was first exposed to anti-V5, then stripped and probed with anti-HA. A Ponceau S stained blot (lower panel) were added to show equal amounts of total proteins loaded onto the gel after isolation from individual strain. Molecular weight marked alongside the marker in kDa.

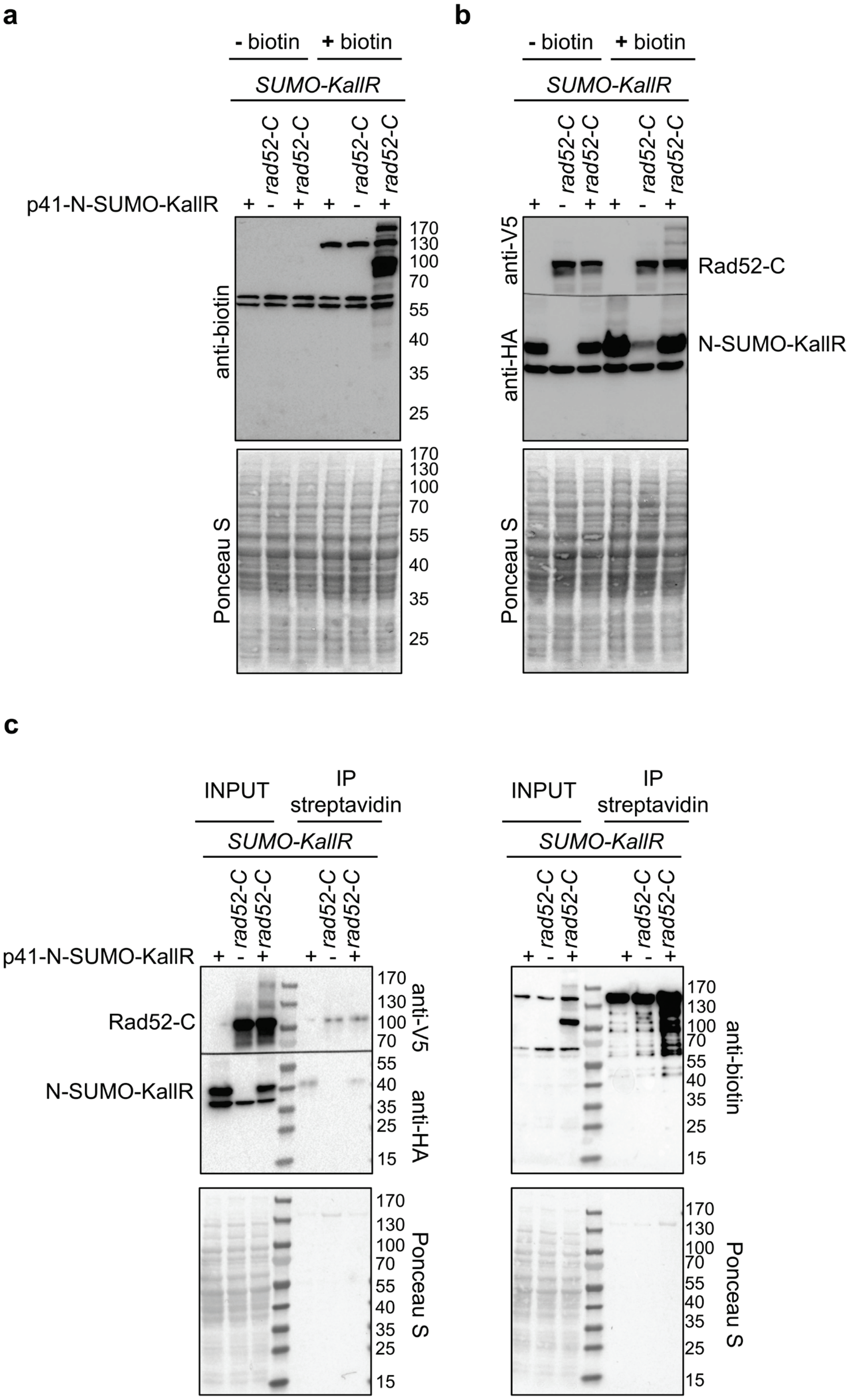

Supplementary Figure 10: Analysis of Rad52- and SUMO-dependent interactome in *SUMO-KallR* background (related to Fig. 4). a Proximal biotinylation assay of proteins by Split-TurboID in indicated transformants and conditions. Western blot analysis was done on total cell extracts prepared from a *SUMO-KallR* mutant and the *SUMO-KallR* Rad52-12Pk-CTurbo (Rad52-C) strains transformed with 6HA-NTurbo-SUMO-KallR (N-SUMO-KallR) using anti-biotin antibody (upper panel). A Ponceau S stained blot

(lower panel) was added to show equal amounts of total proteins loaded onto the gel after isolation from an individual transformant. Molecular weight marked alongside the marker in kDa. **b** Expression of Rad52-12Pk-CTurbo (Rad52-C) and 6HA-NTurbo-SUMO-KallR (N-SUMO-KallR) in indicated transformants and conditions. Western blot analysis with anti-V5 (upper panel upper part), and anti-HA (upper panel bottom part) antibody, respectively, was performed for total protein extracts isolated from exponentially growing cultures of indicated transformants. A Ponceau S stained blot (lower panel) was added to show equal amounts of total proteins loaded onto the gel after isolation from an individual transformant. **c** Streptavidin pulldown of biotinylated proteins in indicated transformants. Same samples were run for Western blot analysis of prepared extracts from a *SUMO-KallR* mutant and the *SUMO-KallR* rad52-12Pk-CTurbo (Rad52-C) mutants transformed with 6HA-NTurbo-SUMO-KallR (N-SUMO-KallR) using anti-biotin (upper right panel), and anti-V5 (upper left panel upper part) or anti-HA (upper left panel bottom part) antibody. A Ponceau S stained blots (lower panels) were added to show amounts of total proteins loaded onto the gel after isolation from individual transformant. INPUTs and IPs on anti-HA and anti-V5 blots are cut and merged from shorter and longer exposures of the same membrane. Molecular weight marked alongside the marker in kDa.

a

WT

	NTurboSUMO_1	NTurboSUMO_2	Rad52CTurbo_1	Rad52CTurbo_2	Split_ID_1	Split_ID_2	Split_ID_3	Group	Sample
		0,91	0,97	0,93	0,82	0,79	0,88	Controls	NTurboSUMO_1
	0,91		0,89	0,96	0,90	0,82	0,94	Controls	NTurboSUMO_2
	0,97	0,89		0,92	0,80	0,77	0,87	Controls	Rad52CTurbo_1
	0,93	0,96	0,92		0,92	0,85	0,98	Controls	Rad52CTurbo_2
	0,82	0,90	0,80	0,92		0,80	0,95	SplitID	Split_ID_1
	0,79	0,82	0,77	0,85	0,80		0,86	SplitID	Split_ID_2
	0,88	0,94	0,87	0,98	0,95	0,86		SplitID	Split_ID_3

0,5 | 0,75 | 1

SUMO-KallR

NTurboSUMO_KallR_1	NTurboSUMO_KallR_2	Rad52CTurbo_1	Rad52CTurbo_2	Split_ID_KallR_1	Split_ID_KallR_2	Split_ID_KallR_3	Group	Sample
	0,84	0,71	0,81	0,84	0,85	0,77	Controls	NTurboSUMO_KallR_1
0,84		0,85	0,91	0,94	0,90	0,91	Controls	NTurboSUMO_KallR_2
0,71	0,85		0,92	0,86	0,87	0,88	Controls	Rad52CTurbo_1
0,81	0,91	0,92		0,93	0,90	0,92	Controls	Rad52CTurbo_2
0,84	0,94	0,86	0,93		0,92	0,95	Split-ID-KallR	Split_ID_KallR_1
0,85	0,90	0,87	0,90	0,92		0,94	Split-ID-KallR	Split_ID_KallR_2
0,77	0,91	0,88	0,92	0,95	0,94		Split-ID-KallR	Split_ID_KallR_3

0,5 | 0,75 | 1

b

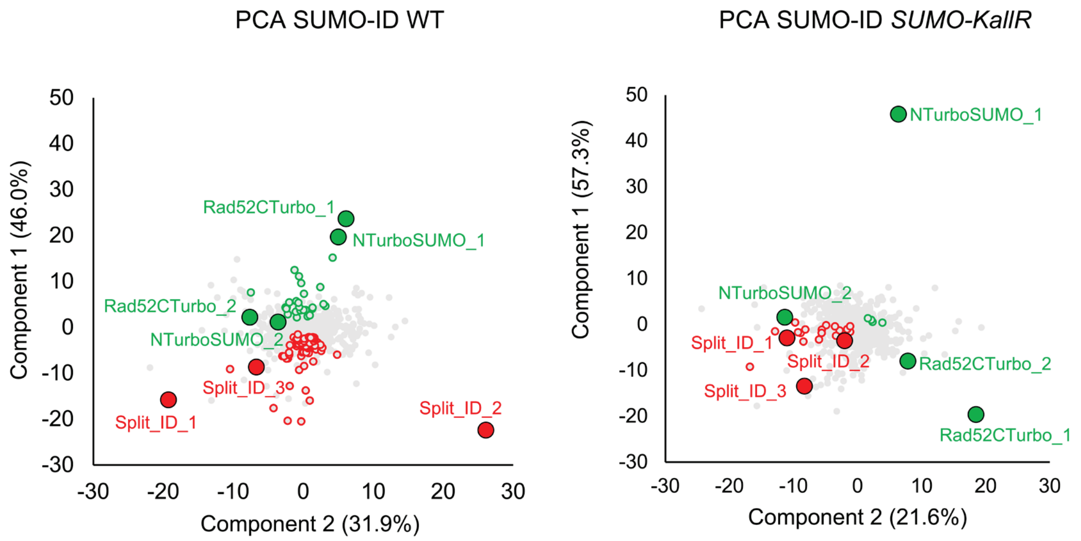

Supplementary Figure 11: Reproducibility analyses (related to Fig. 4). a Protein log2 intensity Pearson correlation coefficient matrix for independent biological replicates of the SUMO-ID (WT) and SUMO-KallR-ID (*SUMO-KallR*) proteomic analyses. **b** Protein log2 intensity principal component analysis plot for independent biological replicates of the SUMO-ID (WT) and SUMO-KallR-ID (*SUMO-KallR*) proteomic analyses. Small gray points indicate each protein's resultant cluster. Large, black-contoured colored

points indicate each sample's resultant cluster. Color-contoured protein clusters indicate proteins for which a significant change was observed in either the Split-ID (red) or the Control (green) group.

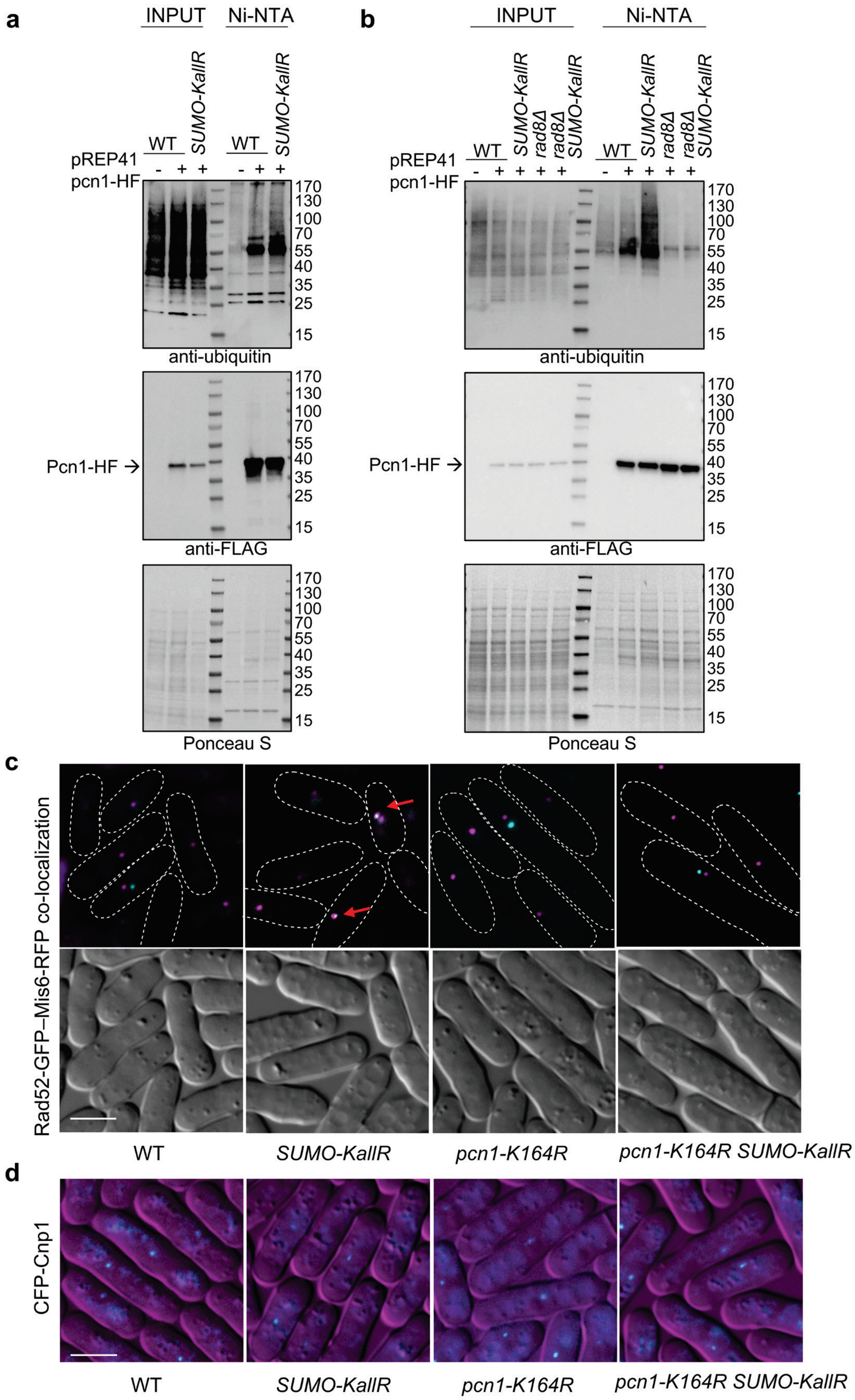

Supplementary Figure 12: Rad8-dependent polyubiquitination of PCNA is increased upon SUMO chains loss (related to Fig. 6). a-b Ni-NTA-pulldown of Pcn1-HF from indicated strains. WT transformed with empty p41 was included as specificity control. Upper panel was probed against ubiquitin, then membranes were stripped and developed against anti-Flag to visualize precipitated Pcn1-HF. Ponceau S is presented to show protein loading. Molecular weight marked alongside the marker in kDa. **c** Examples of live cell imaging of logarithmically growing cells expressing endogenous Rad52-GFP and Mis6-RFP to analyse co-localization between two proteins in indicated strains. Red arrows mark colocalization events. Scale bar = 5 µm. **d** Examples of live cell imaging of logarithmically growing cells expressing endogenous CFP-Cnp1 fusion protein in indicated strains. Scale bar = 5 µm.

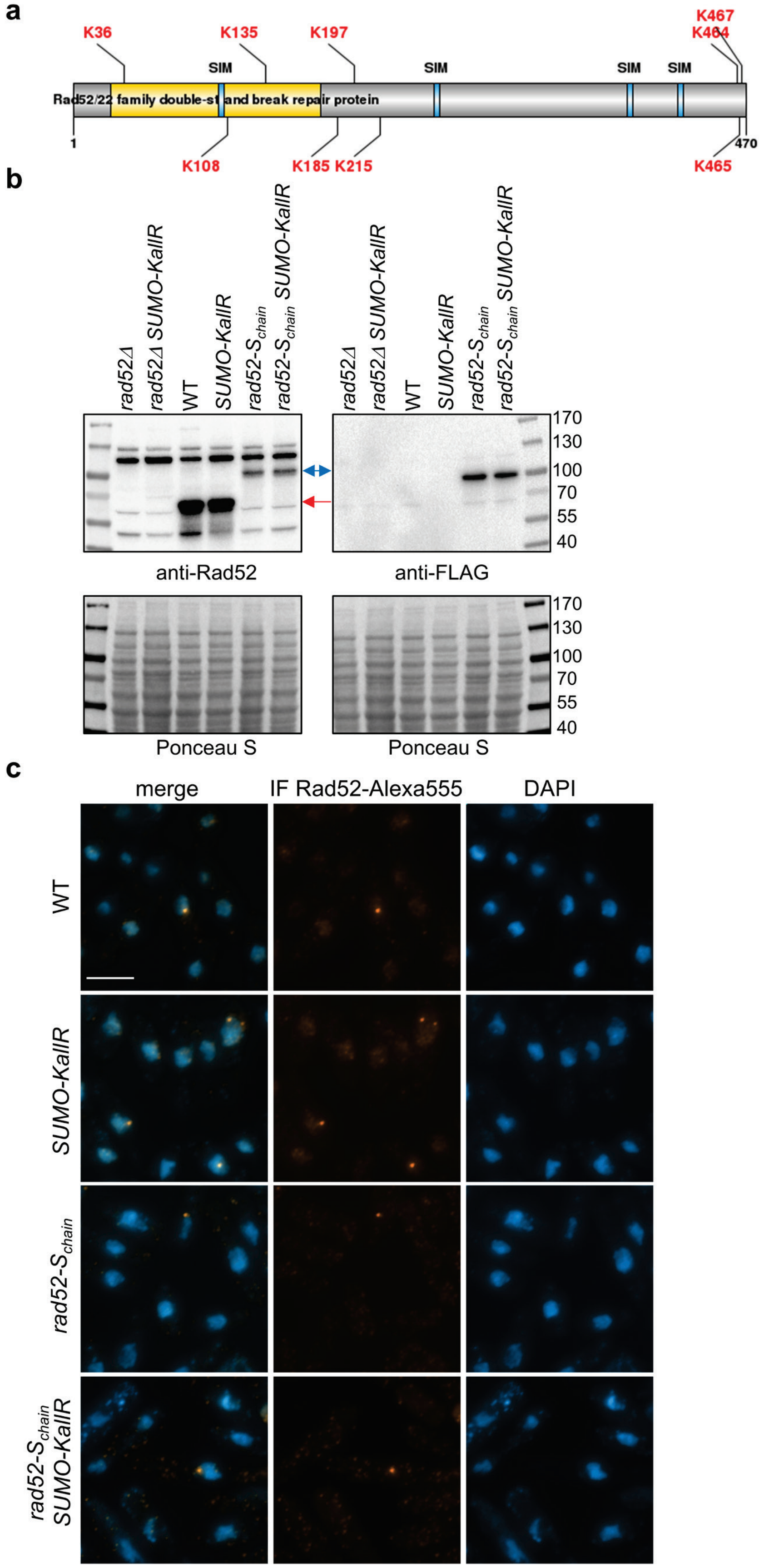

Supplementary Figure 13: PolySUMOylation of Rad52 restrict centromeric recombination (related to Fig. 7). **a** Bioinformatics analysis of Rad52 sequence with GPS SUMO 2.0. Potential SUMOylated lysines and

putative SUMO Interacting Motifs (SIMs) are marked. **b** Western blot analysis showing stable integration of SUMOchain onto endogenous Rad52. Left panel – protein samples developed with anti-Rad52 antibody. Blue arrows mark Rad52-Schain, red arrow marks unmodified Rad52 in control strains. Right panel – same samples developed with anti-Flag antibodies. A Ponceau S stain blot was included to show protein loading. Molecular weight marked alongside the marker in kDa. **c** Example of immunofluorescence images of logarithmic strains containing either Rad52-6His-3Flag (Rad52-HF) or Rad52-SUMOchain-6His-3Flag (Rad52-S_{chain}) probed with anti-Flag (primary) and anti-Rabbit Alexa Fluor 555 (secondary) antibody. Chromatin was stained with DAPI fluorescent dye. Scale bar = 5 µm.

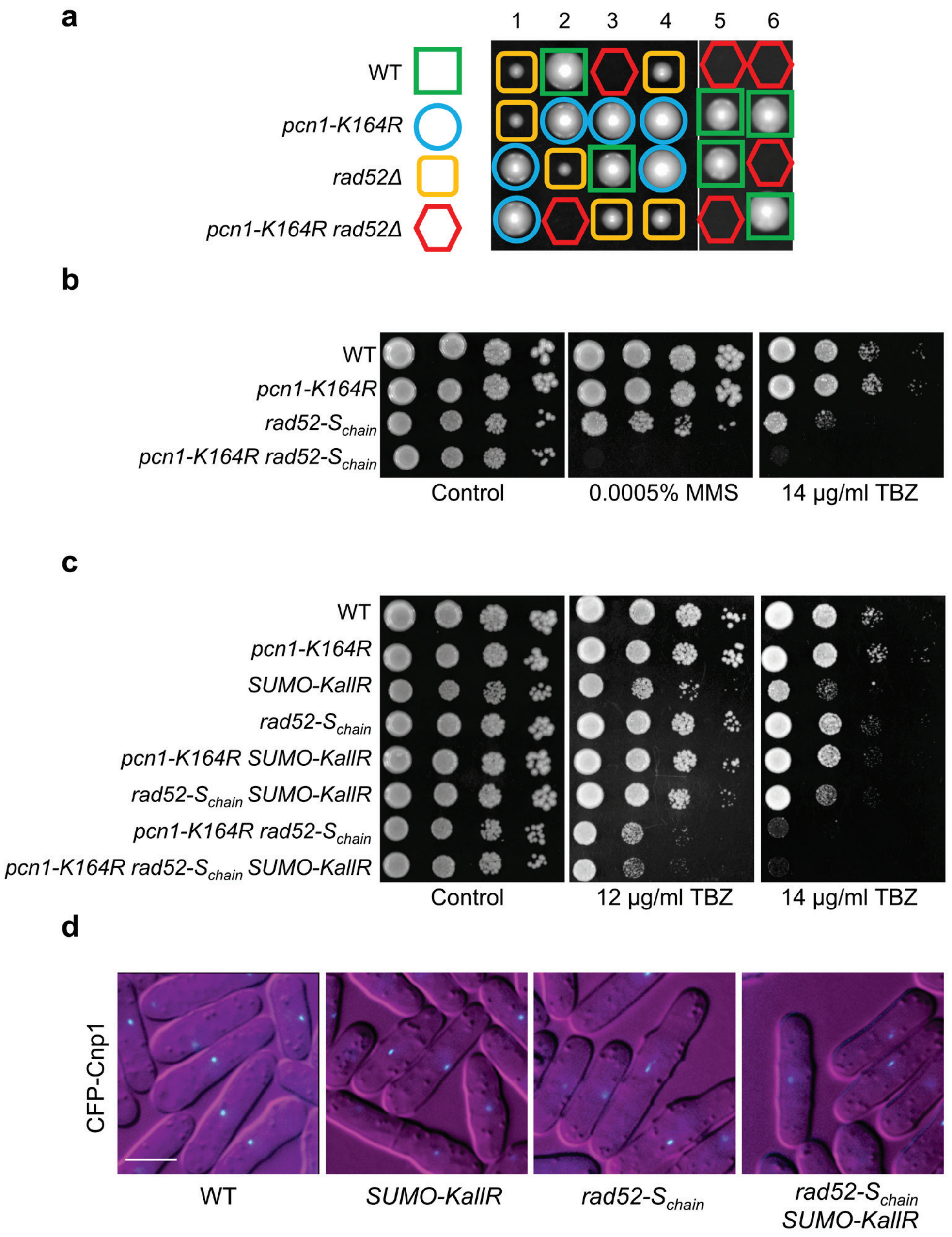

Supplementary Figure 14: Genetic interactions between *pcn1-K164R* and *rad52+* alleles. a Tetrad dissection profile of *pcn1-K164R rad52Δ*. The double mutant is lethal. **b** Analysis of genetic interaction between *pcn1-K164R* and *rad52-S_{chain}*. Ten-fold serial dilution of exponential cultures were dropped on

YES agar plates containing MMS or TBZ in specific concentration. **c** Analysis of genetic interaction between *pcn1-K164R* and *rad52-S*$_{chain}$ in regard of *SUMO-KallR* mutation. Ten-fold serial dilution of exponential cultures were dropped on YES agar plates containing TBZ in specific concentration. **d** Examples of CFP-Cnp1 live cell imaging in indicated mutants. Scale bar = 5 µm.

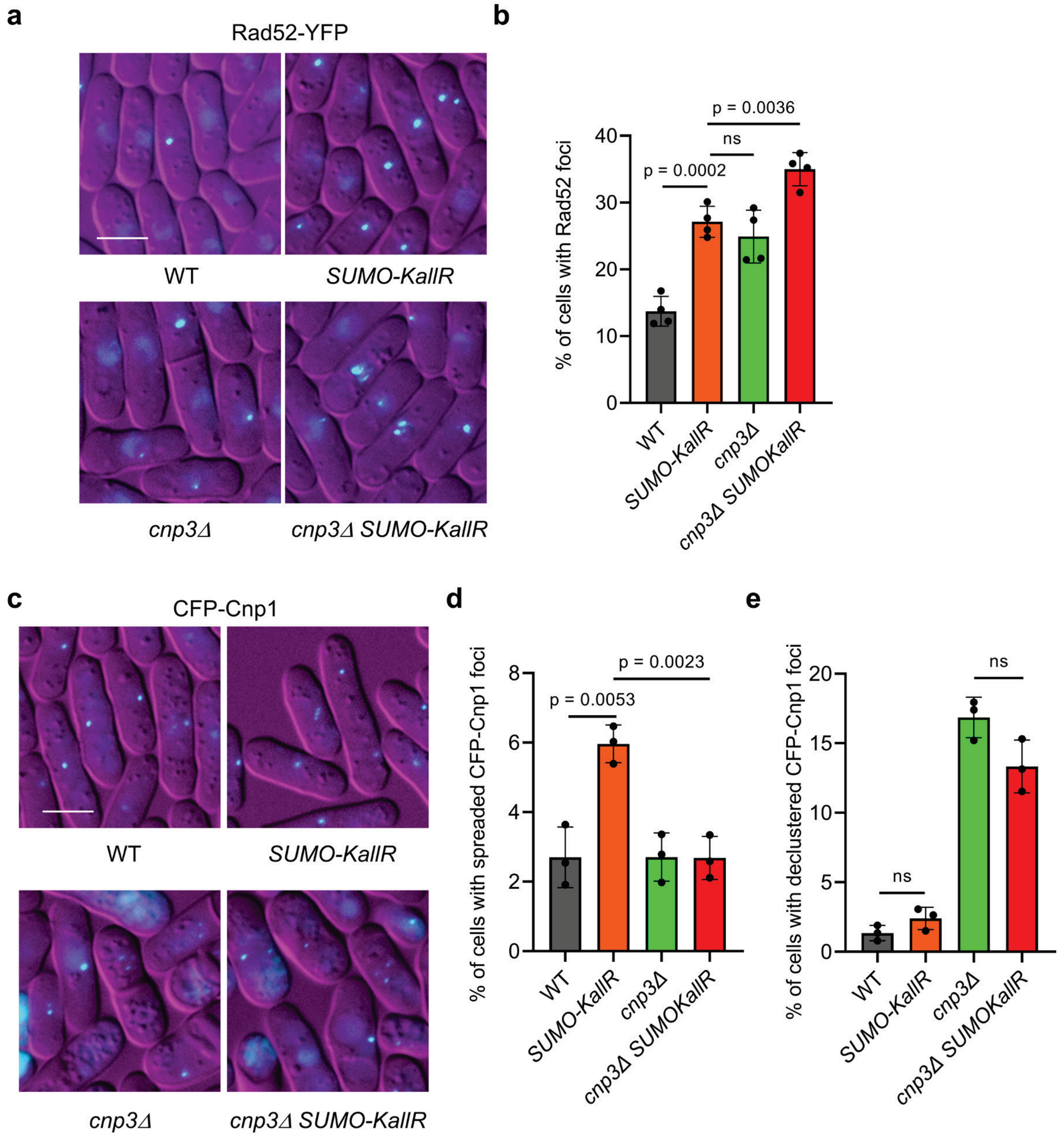

Supplementary Figure 15: Cnp3 inner kinetochore protein might be responsible for disorganization of Cnp1 in *SUMO-KallR* mutant. a Example of fluorescence images of logarithmically growing cells expressing endogenous Rad52-YFP in indicated strains. Scale bar = 5 µm. **b** % of cells forming Rad52-YFP foci in indicated strains. Dots represent values obtained from 4 independent biological experiments. Error bars show the standard deviation (SD) about the mean values. At least 500 cells were analysed for each strain per single experiment. Two-sided Student's *t*-test was used to calculate the *p* values. **c** Examples of CFP-Cnp1 live cell imaging in indicated mutants. Scale bar = 5 µm. **d** % quantification of cells

forming spreaded Cnp1 foci in indicated strains. Dots represent values obtained from 3 independent biological experiments. In all, at least 500 cells were analysed for each strain. Error bars show the SD about the mean values. Two-sided Student's *t*-test was used to calculate the *p* values. **e** % quantification of cells with declustered Cnp1 foci in indicated strains. Dots represent values obtained from 3 independent biological experiments. In all, at least 500 cells were analysed for each strain. Error bars show the SD about the mean values. Two-sided Student's *t*-test was used to calculate the *p* values.

Supplementary Table 1: Strains and plasmids used in this study

Strain number	Genotype	Source
IDN1	*ade6-704 leu1-32 ura4-D18, h+*	S. Lambert
IDN21	*ade6-704 leu1-32 ura4-D18 pmt3::ura4-Kan, h+*	S. Lambert
IDN228	*ade6-704 leu1-32 ura4-D18 pmt3-KallR, h+*	this study
IDN76	*ade6-704 leu1-32 ura4-D18 ssb3-YFP:Nat, h+*	this study
IDN80	*ade6-704 leu1-32 ura4-D18 ssb3-YFP:Nat pmt3::ura4-Kan, h?*	this study
IDN98	*ade6-704 leu1-32 ura4-D18 ssb3-YFP:Nat pmt3-KallR, h?*	this study
IDN67	*ade6-704 leu1-32 ura4-D18 rad52-YFP:Kan, h-*	S. Lambert
IDN95	*ade6-704 leu1-32 ura4-D18 rad52-YFP:Kan pmt3::ura4-Kan, h?*	this study
IDN373	*ade6-704 leu1-32 ura4-D18 rad52-YFP:Kan pmt3-KallR, h?*	this study
IDN134	*ade6-704 his3-D1 leu1-32 ura4-D18 chk1-HA, h?*	D. Dziadkowiec
IDN136	*ade6-704 his3-D1 leu1-32 ura4-D18 chk1-HA pmt3::ura4-Kan, h?*	this study
IDN138	*ade6-704 his3-D1 leu1-32 ura4-D18 chk1-HA pmt3-KallR, h?*	this study
IDN438	*ura4-aim ura4::ADH1-dmNK:Nat-ADH1-hENT1, h+*	S. Lambert
IDN450	*ura4-aim ura4::ADH1-dmNK:Nat-ADH1-hENT1, pmt3-KallR h?*	this study
IDN204	*ade6-704 leu1-32 ura4-D18 rad51::Kan, h?*	S. Lambert
IDN205	*ade6-704 leu1-32 ura4-D18 rad51::Kan pmt3-KallR, h+*	this study
IDN340	*ade6-704 leu1-32 ura4-D18 rad52::Kan, h?*	S. Lambert
IDN229	*ade6-704 leu1-32 ura4-D18 rad52::Kan pmt3-KallR, h+*	this study
IDN310	*ade6-704 leu1-32 ura4-D18 mis6-2mRFP:Hph ssb3-YFP:Nat, h?*	this study
IDN312	*ade6-704 leu1-32 ura4-D18 mis6-2mRFP:Hph ssb3-YFP:Nat pmt3-KallR, h?*	this study
IDN66	*ade6-704 leu1-32 ura4-D18 CFP-cnp1:Kan, h+*	D. Dziadkowiec
IDN104	*ade6-704 leu1-32 ura4-D18 CFP-cnp1:Kan pmt3-KallR, h?*	this study
IDN466	*ade6-704 leu1-32 ura4-D18 slx8-29:Hph*	S. Lambert
IDN467	*ade6-704 leu1-32 ura4-D18 slx8-29:Hph pmt3-KallR*	this study
IDN516	*ade6-704 leu1-32 ura4-D18 CFP-cnp1:Kan slx8-29:Hph*	this study
IDN518	*ade6-704 leu1-32 ura4-D18 CFP-cnp1:Kan slx8-29:Hph pmt3-KallR*	this study

IDN177	*ade6-704 leu1-32 ura4-D18 rad52-YFP:Kan sad1-dsRed:leu2, h-*	this study
IDN197	*ade6-704 leu1-32 ura4-D18 rad52-YFP:Kan sad1-dsRed:leu2 pmt3-KallR, h?*	this study
IDN273	*ade6-704 leu1-32 ura4-D18 loxP:rad51_R152A-R324A-K334A:loxM3, h?*	S. Lambert
IDN274	*ade6-704 leu1-32 ura4-D18 loxP:rad51_R152A-R324A-K334A:loxM3 pmt3-KallR*	this study
IDN386	*ade6-D leu1-32 ura4-D18 imr1L(Hp:ade6B) imr1R(Hp:ade6X), h?*	T. Nakagawa
IDN388	*ade6-D leu1-32 ura4-D18 imr1L(Hp:ade6B) imr1R(Hp:ade6X) pmt3-KallR, h?*	this study
IDN270	*his3-D1 leu1-32 ura4-D18 ade6- L469 int::pUC8/his3+/ade6-M375, h+*	D. Dziadkowiec
IDN292	*his3-D1 leu1-32 ura4-D18 ade6- L469 int::pUC8/his3+/ade6-M375 pmt3-KallR, h?*	this study
IDN198	*ade6-704 leu1-32 ura4-D18 rad52-12Pk-TurboID(79-320):ADH1:Kan, h+*	this study
IDN362	*ade6-704 leu1-32 ura4-D18 rad52-12Pk-TurboID(79-320):ADH1:Kan pmt3-KallR*	this study
IDN69	*ade6-216 his7-366 leu1-32 ura4-DS/E otr1(SphI)::ura4, h+*	S. Lambert
IDN70	*ade6-216 his7-366 leu1-32 ura4-DS/E swi6::Kan otr1(SphI)::ura4, h+*	this study
IDN145	*ade6-216 or ade6-704 leu1-32 ura4-DS/E or ura4-D18 otr1(SphI)::ura4, h?*	this study
IDN148	*ade6-216 or ade6-704 leu1-32 ura4-DS/E or ura4-D18 swi6::Kan otr1(SphI)::ura4, h?*	this study
IDN153	*ade6-216 or ade6-704 leu1-32 ura4-DS/E or ura4-D18 otr1(SphI)::ura4 pmt3-KallR, h?*	this study
IDN154	*ade6-216 or ade6-704 leu1-32 ura4-DS/E or ura4-D18 swi6::Kan otr1(SphI)::ura4 pmt3-KallR, h?*	this study
IDN271	*ade6-DN/N leu1-32 ura4-D18 otr1R(SphI)::ade6, h+*	D. Dziadkowiec
IDN272	*ade6-DN/N leu1-32 ura4-D18 swi6::Nat otr1R(SphI)::ade6, h+*	D. Dziadkowiec
IDN286	*ade6-704 lub ade6-DN/N leu1-32 ura4-D18 otr1R(SphI)::ade6, h?*	this study
IDN288	*ade6-704 lub ade6-DN/N leu1-32 ura4-D18 otr1R(SphI)::ade6 pmt3-KallR, h?*	this study
IDN282	*ade6-704 lub ade6-DN/N leu1-32 ura4-D18 swi6::Nat otr1R(SphI)::ade6, h?*	this study
IDN284	*ade6-704 lub ade6-DN/N leu1-32 ura4-D18 swi6::Nat otr1R(SphI)::ade6 pmt3-KallR, h?*	this study
IDN520	*ade6-704 leu1-32 ura4-D18 pli1::Hph h+*	this study
IDN24	*ade6-704 leu1-32 t-ura4-SD20<ori (RTS1) nmt41:rtf1:sup35 nse2_RING::Hph (deletion of 56 aminoacids from C-terminus) h-*	S. Lambert

IDN470	*ade6-704 leu1-32 ura4-D18 pcn1-K164R h?*	S. Lambert
IDN486	*ade6-704 leu1-32 ura4-D18 pcn1-K164R pmt3-KallR h?*	this study
IDN574	*ade6-704 leu1-32 ura4-D18 rad8::Hph h?*	S. Lambert
IDN575	*ade6-704 leu1-32 ura4-D18 rad8::Hph pmt3-KallR h?*	this study
IDN565	*ade6-704 leu1-32 ura4-D18 rev1::Kan h?*	S. Lambert
IDN566	*ade6-704 leu1-32 ura4-D18 rev1::Kan pmt3-KallR h?*	this study
IDN568	*ade6-704 leu1-32 ura4-D18 rev3::Hph h?*	S. Lambert
IDN569	*ade6-704 leu1-32 ura4-D18 rev3::Hph pmt3-KallR h?*	this study
IDN556	*ade6-D leu1-32 ura4-D18 imr1L(Hp:ade6B) imr1R(Hp:ade6X) pcn1-K164R h?*	this study
IDN557	*ade6-D leu1-32 ura4-D18 imr1L(Hp:ade6B) imr1R(Hp:ade6X) pcn1-K164R pmt3-KallR h?*	this study
IDN607	*ade6-704 leu1-32 ura4-D18 mis6-2mRFP:Hph rad52-GFP:Kan pcn1-K164R h?*	this study
IDN609	*ade6-704 leu1-32 ura4-D18 mis6-2mRFP:Hph rad52-GFP:Kan pcn1-K164R pmt3-KallR h?*	this study
IDN626	*ade6-704 leu1-32 ura4-D18 CFP-cnp1:Kan pcn1-K164R h?*	this study
IDN612	*ade6-704 leu1-32 ura4-D18 CFP-cnp1:Kan pcn1-K164R pmt3-KallR h?*	this study
IDN592	*ade6-704 leu1-32 ura4-D18 rad52-4xpmt3-6his-3flag:Hph h?*	this study
IDN594	*ade6-704 leu1-32 ura4-D18 rad52-4xpmt3-6his-3flag:Hph pmt3-KallR h?*	this study
IDN602	*ade6-D leu1-32 ura4-D18 imr1L(Hp:ade6B) imr1R(Hp:ade6X) rad52-4xpmt3-6his-3flag:Hph h?*	this study
IDN603	*ade6-D ura4-D18 imr1L(Hp:ade6B) imr1R(Hp:ade6X) rad52-4xpmt3-6his-3flag:Hph pmt3-KallR h?*	this study
IDN641	*ade6-704 rad52-6his-3flag:kanMX6 h-*	this study
IDN642	*leu1-32 ura4-D18 rad52-6his-3flag:kanMX6 pmt3-KallR h+*	this study
IDN622	*ade6-704 leu1-32 ura4-D18 CFP-cnp1:Kan rad52-4xpmt3-6his-3flag:Hph h-*	this study
IDN624	*ade6-704 leu1-32 ura4-D18 CFP-cnp1:Kan rad52-4xpmt3-6his-3flag:Hph pmt3-KallR h-*	this study
IDN586	*ade6-704 lub ade6-M216 leu1-32 ura4-D18 cnp3::Kan rad52-YFP:Kan h?*	this study
IDN588	*ade6-704 lub ade6-M216 leu1-32 ura4-D18 cnp3::Kan rad52-YFP:Kan pmt3-KallR h?*	this study
IDN613	*ade6-704 lub ade6-M216 leu1-32 ura4-D18 CFP-cnp1:Kan cnp3::Kan h?*	this study
IDN614	*ade6-704 lub ade6-M216 leu1-32 ura4-D18 CFP-cnp1:Kan cnp3::Kan pmt3-KallR h?*	this study
IDN660	*ade6-704 leu1-32 ura4-D18 rad52-6his-3flag:kanMX6 pli1::Hph h?*	this study
IDN670	*leu1-32 ura4-D18 nmt41:rtf1:sup35 rad52-6his-3flag:kanMX6 nse2_RING::Hph h?*	this study

IDN677	*ade6-704 leu1-32 ura4-D18 pcn1-K164R rad52-4xpmt3-6his-3flag:Hph h?*	this study
IDN678	*ade6-704 leu1-32 ura4-D18 pcn1-K164R rad52-4xpmt3-6his-3flag:Hph pmt3-KallR h?*	this study
JC1315	*MATa ade2-1 can1-100 ura3-1 his3-11,15 leu2-3,112 trp1-1 RAD5 SCC1-9PK-TRP1 (S. cerevisiae)*	J. Cobb
IDN_P13	*pREP41-MSC+*	Addgene 52690
IDN_P56	*pREP41-nmt41-6HA-TurboID(1-78)-pmt3*	this study
IDN_P75	*pREP41-nmt41-6HA-NTurboID(1-78)-pmt3-KallR*	this study
IDN_P67	*pFA6a-12Pk-CTurbo (79-320)-KanMX6*	this study
IDN_P74	*pREP41-nmt41-6his-3flag-pcn1*	this study
IDN_P82	*pREP41-nmt41-6his-3flag-pcn1-K164R*	this study
IDN_P85	*pREP41-3flag*	this study
IDN_P77	*pFA6a-4xpmt3-6his-3flag-hphMX6*	this study

Supplementary Table 2: Primers used in this study

Primer name	Sequence (5'-3')	Experiment
II50_F	CACCGCAGTTCTACGTATCCT	ChIP (Control locus on ChrII)
II50_R	CGATGTAACGGTATGCGGTA	ChIP (Control locus on ChrII)
cnt_F	CAACCGTTGCAACTTACATCAGCA	CFP-cnp1, Rad52-YFP, and Rad51 ChIP
cnt_R	CCGGTCGCCAAATAGCAATGAGAT	CFP-cnp1, Rad52-YFP, and Rad51 ChIP
dg_F	TACCGTGATTAGCCTTACTCCGCA	CFP-cnp1, Rad52-YFP, and Rad51 ChIP
dg_R	ACCGCAAGATAGAGTAGGATGGGT	CFP-cnp1, Rad52-YFP, and Rad51 ChIP
act1_F	CGCCGAACGTGAAATTGTTCGTGA	ChIP (Control locus)
act1_R	AAGGGAGGAAGATTGAGCAGCAGT	ChIP (Control locus)
qPCR_ura4_F	CTCTTTGGCTACTGGTTCCTAC	*otr1(SphI)::ura4* transcription
qPCR_ura4_R	GGAAATCGACGACCAGCTATAA	*otr1(SphI)::ura4* transcription
ura4-DS/E_F	GGCTCTTTGGCTACTGGTTC	endogenous *ura4* deletion check
ura4-DS/E_R	GGGACATAGTTATGTAGTCGC	endogenous *ura4* deletion check
