# Supplementary material for: PolySUMOylation of PCNA and Rad52 restricts centromeric recombination in fission yeast
Source: Nat Commun. 2025 Dec 2;16:10837. doi: 10.1038/s41467-025-65862-1 (PMC12672585; doi:10.1038/s41467-025-65862-1)
Supplement: Supplementary file 2 — Description of Additonal Supplementary Files [file 41467_2025_65862_MOESM2_ESM.pdf]

Description of Additional Supplementary Files

Supplementary Data 1

Description: All data generated by mass spectrometry SUMO-ID assay for WT and SUMO-KallR background. Description of exact sheets included within the Excel file.
