# Supplementary material for: PolySUMOylation of PCNA and Rad52 restricts centromeric recombination in fission yeast
Source: Nat Commun. 2025 Dec 2;16:10837. doi: 10.1038/s41467-025-65862-1 (PMC12672585; doi:10.1038/s41467-025-65862-1)
Supplement: Supplementary file 4 — Reporting Summary [file 41467_2025_65862_MOESM4_ESM.pdf]

nature portfolio

Corresponding author(s): Karol Kramarz

Last updated by author(s): Oct 17, 2025

Reporting Summary

Nature Portfolio wishes to improve the reproducibility of the work that we publish. This form provides structure for consistency and transparency in reporting. For further information on Nature Portfolio policies, see our Editorial Policies and the Editorial Policy Checklist.

Statistics

For all statistical analyses, confirm that the following items are present in the figure legend, table legend, main text, or Methods section.

n/a	Confirmed	
☐	☒	The exact sample size (*n*) for each experimental group/condition, given as a discrete number and unit of measurement
☐	☒	A statement on whether measurements were taken from distinct samples or whether the same sample was measured repeatedly
☐	☒	The statistical test(s) used AND whether they are one- or two-sided<br>*Only common tests should be described solely by name; describe more complex techniques in the Methods section.*
☒	☐	A description of all covariates tested
☒	☐	A description of any assumptions or corrections, such as tests of normality and adjustment for multiple comparisons
☐	☒	A full description of the statistical parameters including central tendency (e.g. means) or other basic estimates (e.g. regression coefficient) AND variation (e.g. standard deviation) or associated estimates of uncertainty (e.g. confidence intervals)
☐	☒	For null hypothesis testing, the test statistic (e.g. *F*, *t*, *r*) with confidence intervals, effect sizes, degrees of freedom and *P* value noted<br>*Give P values as exact values whenever suitable.*
☒	☐	For Bayesian analysis, information on the choice of priors and Markov chain Monte Carlo settings
☒	☐	For hierarchical and complex designs, identification of the appropriate level for tests and full reporting of outcomes
☐	☒	Estimates of effect sizes (e.g. Cohen's *d*, Pearson's *r*), indicating how they were calculated

Our web collection on statistics for biologists contains articles on many of the points above.

Software and code

Policy information about availability of computer code

Data collection	ZEN Microscope Software, Bio-Rad Maestro CFX 1.1, Bio-rad Image-Lab (for Chemidoc MP), InCyte 2.7 (for Guava easyCyte flow cytometry), MassLynx v4.2 (Waters) for mass spectrometry data acquisition
Data analysis	ImageJ, Graphpad Prism 9, MS Office, Corel Draw, Python, Progenesis QiP v4.2.7 for mass spectrometry data processing and analysis, Perseus for mass spectrometry data statistical analysis

For manuscripts utilizing custom algorithms or software that are central to the research but not yet described in published literature, software must be made available to editors and reviewers. We strongly encourage code deposition in a community repository (e.g. GitHub). See the Nature Portfolio guidelines for submitting code & software for further information.

Data

Policy information about availability of data

All manuscripts must include a data availability statement. This statement should provide the following information, where applicable:

- Accession codes, unique identifiers, or web links for publicly available datasets
- A description of any restrictions on data availability
- For clinical datasets or third party data, please ensure that the statement adheres to our policy

ChIP-seq dataset was deposited to GEO: GSE276805, public access
Mass spectrometry data were deposited to PRIDE under accession numbers: project doi: 10.6019/PXD055556, reviewer login: reviewer_pxd055556@ebi.ac.uk, password: 6z4ZZgJ0tmYG

The source data for all main figures and supplementary figures are provided as a Source Data file in MS Excel. Spreadsheets for all the graphical representations are submitted as source data in excel files (.xls format).
All relevant data are available and further information and requests for reagents and resources will be fulfilled by Dr. Karol Kramarz (karol.kramarz@uwr.edu.pl).

Research involving human participants, their data, or biological material

Policy information about studies with human participants or human data. See also policy information about sex, gender (identity/presentation), and sexual orientation and race, ethnicity and racism.

Reporting on sex and gender	*Use the terms sex (biological attribute) and gender (shaped by social and cultural circumstances) carefully in order to avoid confusing both terms. Indicate if findings apply to only one sex or gender; describe whether sex and gender were considered in study design; whether sex and/or gender was determined based on self-reporting or assigned and methods used. Provide in the source data disaggregated sex and gender data, where this information has been collected, and if consent has been obtained for sharing of individual-level data; provide overall numbers in this Reporting Summary. Please state if this information has not been collected. Report sex- and gender-based analyses where performed, justify reasons for lack of sex- and gender-based analysis.*
Reporting on race, ethnicity, or other socially relevant groupings	*Please specify the socially constructed or socially relevant categorization variable(s) used in your manuscript and explain why they were used. Please note that such variables should not be used as proxies for other socially constructed/relevant variables (for example, race or ethnicity should not be used as a proxy for socioeconomic status). Provide clear definitions of the relevant terms used, how they were provided (by the participants/respondents, the researchers, or third parties), and the method(s) used to classify people into the different categories (e.g. self-report, census or administrative data, social media data, etc.) Please provide details about how you controlled for confounding variables in your analyses.*
Population characteristics	*Describe the covariate-relevant population characteristics of the human research participants (e.g. age, genotypic information, past and current diagnosis and treatment categories). If you filled out the behavioural & social sciences study design questions and have nothing to add here, write "See above."*
Recruitment	*Describe how participants were recruited. Outline any potential self-selection bias or other biases that may be present and how these are likely to impact results.*
Ethics oversight	*Identify the organization(s) that approved the study protocol.*

Note that full information on the approval of the study protocol must also be provided in the manuscript.

Field-specific reporting

Please select the one below that is the best fit for your research. If you are not sure, read the appropriate sections before making your selection.

☒ Life sciences ☐ Behavioural & social sciences ☐ Ecological, evolutionary & environmental sciences

For a reference copy of the document with all sections, see nature.com/documents/nr-reporting-summary-flat.pdf

Life sciences study design

All studies must disclose on these points even when the disclosure is negative.

Sample size	No statistical methods were utilized to predetermine sample size, because this study did not include animal models or human participants. Sample size was based on standards in the field and to obtain statistical significance and reproducibility, typically at least three biological replicates were performed.
Data exclusions	No data were excluded from analysis.
Replication	Most of experimental findings were reproduced three times and are presented as single values for all the plots. ChIP-seq analysis of CFP-Cnp1 binding across chromosome was done once, because obtained readouts were robust and the profile obtained by NGS was confirmed by standard ChIP-qPCR analysis and live cell imaging of CFP-Cnp1.
Randomization	No randomization was done. This study did not involve animals or human participants. Samples were grouped based on treatment and genotype. Relevant controls were included in all experiments.
Blinding	No blinded group allocation, because of the nature of biological samples and type of experiments performed.

Reporting for specific materials, systems and methods

We require information from authors about some types of materials, experimental systems and methods used in many studies. Here, indicate whether each material, system or method listed is relevant to your study. If you are not sure if a list item applies to your research, read the appropriate section before selecting a response.

Materials & experimental systems

n/a	Involved in the study
☐	☒ Antibodies
☒	☐ Eukaryotic cell lines
☒	☐ Palaeontology and archaeology
☒	☐ Animals and other organisms
☒	☐ Clinical data
☒	☐ Dual use research of concern
☒	☐ Plants

Methods

n/a	Involved in the study
☐	☒ ChIP-seq
☐	☒ Flow cytometry
☒	☐ MRI-based neuroimaging

Antibodies

Antibodies used

Antibodies were used as follows:

Anti-SUMO (Pmt3), non-commercial, polyclonal, produced by Agro-Bio company in rabbit, a gift from Dr. Sarah Lambert, dilution for WB: 1:2000
Anti-ubiquitin (P4D1, Santa Cruz, sc-8017) WB dilution 1:500
Anti-Flag (Sigma-Aldrich, F7425) WB dilution: 1:2000, IF 1:300
Anti-Rad51 (Abcam, ab63799) WB dilution 1:5000, IF 1:300, ChIP:1:150
Anti-GFP (Invitrogen A11122) ChIP 1:150, WB 1:1000
Anti-H3 (Abcam, ab1791) WB 1:1000
Anti-HA (Sigma-Aldrich, H6908) WB 1:3000
Anti-PK/V5 (Bio-Rad, MCA1360G) WB: 1:3000
Anti-biotin (Rockland 200-301-098) WB: 1:3000
Anti-Rad52 (BioAcademia, 63-003) WB 1:3000
Normal Rabbit IgG (Cell signaling technology, 2729) ChIP 1:150
anti-Rabbit Alexa Fluor 555 (Invitrogen, A21428) IF: 1:2000

Validation

Antibodies were validated against strains that do not expressed relevant epitopes, where applicable.

anti-SUMO: Schirmeisen K, Naiman K, Fréon K, Besse L, Chakraborty S, Saada AA, Carr AM, Kramarz K, Lambert SAE. SUMO protease and proteasome recruitment at the nuclear periphery differently affect replication dynamics at arrested forks. Nucleic Acids Res. 2024 doi: 10.1093/nar/gkae526.

anti-ubiquitin: Chen ZJ, Parent L, Maniatis T. Site-specific phosphorylation of IkappaBalpha by a novel ubiquitination-dependent protein kinase activity. Cell. 1996 84(6):853-62. doi: 10.1016/s0092-8674(00)81064-8

anti-Flag: Koumoundourou A, Rannap M, De Bruyckere E, Nestel S, Reissner C, Egorov AV, Liu P, Missler M, Heimrich B, Draguhn A, Britsch S. Regulation of hippocampal mossy fiber-CA3 synapse function by a Bcl11b/C1ql2/Nrxn3(25b+) pathway. Elife. 2024 Feb 15;12:RP89854. doi: 10.7554/eLife.89854. PMID: 38358390; PMCID: PMC10942602.

anti-Rad51: Telomerase Repairs Collapsed Replication Forks at Telomeres. Cell Rep. 2020 Mar 10;30(10):3312-3322.e3. doi: 10.1016/j.celrep.2020.02.065. PMID: 32160539.

anti-GFP: Choi ES, Cheon Y, Kang K, Lee D. The Ino80 complex mediates epigenetic centromere propagation via active removal of histone H3. Nat Commun. 2017 Sep 13;8(1):529. doi: 10.1038/s41467-017-00704-3. PMID: 28904333; PMCID: PMC5597579.

anti-H3: Lee HS, Bang I, You J, Jeong TK, Kim CR, Hwang M, Kim JS, Baek SH, Song JJ, Choi HJ. Molecular basis for PHF7-mediated ubiquitination of histone H3. Genes Dev. 2023 Dec 26;37(21-24):984-997. doi: 10.1101/gad.350989.123. PMID: 37993255; PMCID: PMC10760634.

anti-HA: O'Donovan KJ, Diedler J, Couture GC, Fak JJ, Darnell RB. The onconeural antigen cdr2 is a novel APC/C target that acts in mitosis to regulate c-myc target genes in mammalian tumor cells. PLoS One. 2010 Apr 7;5(4):e10045. doi: 10.1371/journal.pone.0010045.

anti-PK/V5: Ng MY, Wang M, Casey PJ, Gan YH, Hagen T. Activation of MAPK/ERK signaling by Burkholderia pseudomallei cycle inhibiting factor (Cif). PLoS One. 2017 Feb 6;12(2):e0171464. doi: 10.1371/journal.pone.0171464.

anti-biotin: George J, Li Y, Kadamberi IP, Parashar D, Tsaih SW, Gupta P, Geethadevi A, Chen C, Ghosh C, Sun Y, Mittal S, Ramchandran R, Rui H, Lopez-Berestein G, Rodriguez-Aguayo C, Leone G, Rader JS, Sood AK, Dey M, Pradeep S, Chaluvally-Raghavan P. RNA-binding protein FXR1 drives cMYC translation by recruiting eIF4F complex to the translation start site. Cell Rep. 2021 Nov 2;37(5):109934. doi: 10.1016/j.celrep.2021.109934. Erratum in: Cell Rep. 2023 Mar 28;42(3):112228. doi: 10.1016/j.celrep.2023.112228.

anti-Rad52: Kishkevich A, Tamang S, Nguyen MO, Oehler J, Bulmaga E, Andreadis C, Morrow CA, Jalan M, Osman F, Whitby MC. Rad52's DNA annealing activity drives template switching associated with restarted DNA replication. Nat Commun. 2022 Nov 26;13(1):7293. doi: 10.1038/s41467-022-35060-4.

Normal Rabbit IgG: Hu H, Ji Q, Song M, Ren J, Liu Z, Wang Z, Liu X, Yan K, Hu J, Jing Y, Wang S, Zhang W, Liu GH, Qu J. ZKSCAN3 counteracts cellular senescence by stabilizing heterochromatin. Nucleic Acids Res. 2020 Jun 19;48(11):6001-6018. doi: 10.1093/nar/gkaa425

anti-Rabbit Alexa Fluor 555: Liang K, Zhang M, Liang J, Zuo X, Jia X, Shan J, Li Z, Yu J, Xuan Z, Luo L, Zhao H, Gan S, Liu D, Qin Q, Wang Q. M1-type polarized macrophage contributes to brain damage through CXCR3.2/CXCL11 pathways after RGNNV infection in grouper. Virulence. 2024 Dec;15(1):2355971. doi: 10.1080/21505594.2024.2355971.

Plants

Seed stocks

Report on the source of all seed stocks or other plant material used. If applicable, state the seed stock centre and catalogue number. If plant specimens were collected from the field, describe the collection location, date and sampling procedures.

Novel plant genotypes

Describe the methods by which all novel plant genotypes were produced. This includes those generated by transgenic approaches, gene editing, chemical/radiation-based mutagenesis and hybridization. For transgenic lines, describe the transformation method, the number of independent lines analyzed and the generation upon which experiments were performed. For gene-edited lines, describe the editor used, the endogenous sequence targeted for editing, the targeting guide RNA sequence (if applicable) and how the editor was applied.

Authentication

Describe any authentication procedures for each seed stock used or novel genotype generated. Describe any experiments used to assess the effect of a mutation and, where applicable, how potential secondary effects (e.g. second site T-DNA insertions, mosiacism, off-target gene editing) were examined.

ChIP-seq

Data deposition

☒ Confirm that both raw and final processed data have been deposited in a public database such as GEO.

☒ Confirm that you have deposited or provided access to graph files (e.g. BED files) for the called peaks.

Data access links
May remain private before publication.

https://www.ncbi.nlm.nih.gov/geo/query/acc.cgi?acc=GSE276805

Files in database submission

RAW FILES
file name
untagged_IP_R1.fq.gz
untagged_INP_R1.fq.gz
WT_CFP-cnp1_IP_R1.fq.gz
WT_CFP-cnp1_INP_R1.fq.gz
SUMO-KallR_CFP-cnp1_IP_R1.fq.gz
SUMO-KallR_CFP-cnp1_INP_R1.fq.gz
untagged_IP_R2.fq.gz
untagged_INP_R2.fq.gz
WT_CFP-cnp1_IP_R2.fq.gz
WT_CFP-cnp1_INP_R2.fq.gz
SUMO-KallR_CFP-cnp1_IP_R2.fq.gz
SUMO-KallR_CFP-cnp1_INP_R2.fq.gz

PROCESSED DATA FILES
file name
untagged_IP_pvalue.bw
untagged_INP.bigWig
WT_CFP-cnp1_IP_pvalue.bw
WT_CFP-cnp1_INP.bigWig
SUMO-KallR_CFP-cnp1_IP_pvalue.bw
SUMO-KallR_CFP-cnp1_INP.bigWig
untagged_IP_pvalue.narrowPeak
WT_CFP-cnp1_IP_pvalue.narrowPeak
SUMO-KallR_CFP-cnp1_IP_pvalue.narrowPeak

SRA
untagged, ChIP: SRX26039752
untagged, Input: SRX26039753
WT_CFP-cnp1, ChIP: SRX26039754
WT_CFP-cnp1, Input: SRX26039755
SUMO-KallR_CFP-cnp1, ChIP: SRX26039756
SUMO-KallR_CFP-cnp1, Input: SRX26039757

Genome browser session (e.g. UCSC) | no longer applicable

Methodology

Replicates

Number of replicates: 1. We are working on a small fission yeast genome which means the sequencing depth is very high. The ChIP-seq data are supported by other experimental results, including standard ChIP-qPCR on CFP-Cnp1 and fluorescent microscopy of CFP-Cnp1.

Sequencing depth

Total number of reads: 21543648 (untagged_IP_R1.fq.gz); 21543648 (untagged_IP_R2.fq.gz); 15562127 (untagged_INP_R1.fq.gz); 15562127 (untagged_INP_R2.fq.gz); 17222354 (WT_CFP-cnp1_IP_R1.fq.gz); 17222354 (WT_CFP-cnp1_IP_R2.fq.gz); 19819382 (WT_CFP-cnp1_INP_R1.fq.gz); 19819382 (WT_CFP-cnp1_INP_R1.fq.gz); 21037023 (SUMO-KallR_CFP-cnp1_IP_R1.fq.gz); 21037023 (SUMO-KallR_CFP-cnp1_IP_R2.fq.gz); 13809408 (SUMO-KallR_CFP-cnp1_INP_R1.fq.gz); 13809408 (SUMO-KallR_CFP-cnp1_INP_R2.fq.gz)

Uniquely mapped reads to S. pombe: 20634370 (untagged_IP); 16273787 (untagged_INP); 16166467 (WT_CFP-cnp1_IP); 19874842 (WT_CFP-cnp1_INP); 20908005 (SUMO-KallR_CFP-cnp1_IP); 15567253 (SUMO-KallR_CFP-cnp1_INP)

Length of reads: 150bp

Paired-end

Antibodies

anti-GFP antibody (Invitrogen, A11122)

Peak calling parameters

macs3 callpeak --keep-dup all --mfold 1 50 --gsize 12590849 --nomodel --extsize 200 --format BAM -t IP.bam -c INP.bam

Data quality

Peaks: 961 (untagged_IP); 59 (WT_CFP-cnp1_IP); 1615 (SUMO-KallR_CFP-cnp1_IP)

Software

Raw sequencing data was quality controlled by Q30. Low-quality reads and adapter sequences were cut off using Cutadapt. Filtered reads were then mapped against the target genome (S. pombe assembly ASM294v2) and against the spike-in genome (S. cerevisiae S288C assembly SacCer3) using Bowtie2. Unmapped reads, rDNA regions, mitochondrial DNA and duplicates were filtered out using Samtools. Samtools was also used to count reads uniquely mapped to ASM294v2 and SacCer3, and these values were used to calculate the occupancy ratio value. Using MACS3, we computed the local bias by taking the maximum bias from surrounding 1kb, 10kb, the size of fragment length d, and the whole genome background. Finally, we combined and generated the maximum background noise, and scaled the ChIP and control to the same sequencing depth. Spike-in calibration was performed according to Hu et al. (2015). Peaks were called using MACS3 bdgpeakcall function. bigWig files were created using deepTools.

Flow Cytometry

Plots

Confirm that:

- [x] The axis labels state the marker and fluorochrome used (e.g. CD4-FITC).
- [x] The axis scales are clearly visible. Include numbers along axes only for bottom left plot of group (a 'group' is an analysis of identical markers).
- [x] All plots are contour plots with outliers or pseudocolor plots.
- [ ] A numerical value for number of cells or percentage (with statistics) is provided.

Methodology

Sample preparation

Flow cytometry analysis of DNA content was performed as follows: cells were fixed in 70 % ethanol and washed with 50 mM sodium citrate, digested with RNAse A (Sigma-Aldrich, R5503) for 2h, stained with 1 µM Sytox Green nucleic acid stain (Invitrogen, S7020) and subjected to flow cytometry using Guava easyCyte flow cytometer (Millipore).

Instrument

Guava easyCyte flow cytometer (Millipore)

Software

InCyte 2.7

Cell population abundance

5000 events/sample

Gating strategy

Forward and side scatter paramters (FSC and SSC) were verified to exclude cell debris and dublets as shown in Supplementary Figure 2a. Overall, less than 4% of cell population was excluded to remove noise signal at the boundaries of histograms of DNA content

- [x] Tick this box to confirm that a figure exemplifying the gating strategy is provided in the Supplementary Information.
